# Prediction of osteoporosis from proximal femoral cortical bone thickness and Hounsfield unit value with clinical significance

**DOI:** 10.3389/fsurg.2022.1047603

**Published:** 2023-01-06

**Authors:** Gaoxiang Xu, Daofeng Wang, Hao Zhang, Cheng Xu, Hua Li, Wupeng Zhang, Jiantao Li, Licheng Zhang, Peifu Tang

**Affiliations:** ^1^ Medical School of Chinese PLA, Chinese PLA General Hospital, Beijing, China; ^2^ Department of Orthopedics, The Fourth Medical Center, Chinese PLA General Hospital, Beijing, China; ^3^ National Clinical Research Center for Orthopedics, Sports Medicine and Rehabilitation, Chinese PLA General Hospital, Beijing, China; ^4^ School of Medicine, Nankai University, Tianjin, China

**Keywords:** osteoporosis, bone mineral density, proximal femur, cortical bone thickness, Hounsfield unit

## Abstract

**Background:**

Utilizing dual-energy x-ray absorptiometry (DXA) to assess bone mineral density (BMD) was not routine in many clinical scenarios, leading to missed diagnoses of osteoporosis. The objective of this study is to obtain effective parameters from hip computer tomography (CT) to screen patients with osteoporosis and predict their clinical outcomes.

**Methods:**

A total of 375 patients with hip CT scans for intertrochanteric fracture were included. Among them, 56 patients possessed the data of both hip CT scans and DXA and were settled as a training group. The cortical bone thickness (CTh) and Hounsfield unit (HU) values were abstracted from 31 regions of interest (ROIs) of the proximal femur. In the training group, the correlations between these parameters and BMD were investigated, and their diagnostic efficiency of osteoporosis was assessed. Finally, 375 patients were divided into osteoporotic and nonosteoporotic groups based on the optimal cut-off values, and the clinical difference between subgroups was evaluated.

**Results:**

The CTh value of ROI 21 and the HU value of ROI 14 were moderately correlated with the hip BMD [*r* = 0.475 and 0.445 (*p* < 0.001), respectively]. The best diagnostic effect could be obtained by defining osteoporosis as CTh value < 3.19 mm in ROI 21 or HU value < 424.97 HU in ROI 14, with accuracies of 0.821 and 0.883, sensitivities of 84% and 76%, and specificities of 71% and 87%, respectively. The clinical outcome of the nonosteoporotic group was better than that of the osteoporotic group regardless of the division criteria.

**Conclusion:**

The CTh and HU values of specific cortex sites in the proximal femur were positively correlated with BMD of DXA at the hip. Thresholds for osteoporosis based on CTh and HU values could be utilized to screen osteoporosis and predict clinical outcomes.

## Introduction

Osteoporosis, an age-related illness marked by reduced bone mineral density (BMD) and microarchitectural deterioration, could lead to a series of osteoporotic fractures (1). In the global population over 50 years old, 33% of women and 20% of men would encounter one or more osteoporotic fractures (2). The mortality rate within 1 year was as high as 20%, and the permanent disability rate was as high as 50% (3). Meanwhile, due to reduced bone strength, the incidence of postoperative complications of osteoporotic fractures was significantly higher than that of nonosteoporotic fractures (4, 5). These cost the China healthcare system approximately 18.9 billion dollars annually (6). Thus, an early screening of osteoporosis would be advantageous to allocate considerable resources for preventing osteoporotic fractures and postoperative complications without exceeding those incurred following these problems (7).

Dual-energy x-ray absorptiometry (DXA) and quantitative computed tomography (QCT) have been applied to identify osteoporosis as the gold standard (8). BMD assessed in exact areas of the hip or lumbar spine was commonly utilized to evaluate bone health and fracture risk (9). However, the lack of both tests in primary medical institutions, the untimely examination of patients in senior medical institutions, and the additional medical costs resulting from routine examination make many patients easily missed. Therefore, how to apply the existing imaging data to screen osteoporosis to avoid the occurrence of osteoporotic fractures and postoperative complications has attracted high attention of researchers.

Cortical thickness could be more easily acquired in routine orthopedic radiographic examinations, and this value in the proximal femur has been proven to be correlated with BMD and of ability to predict osteoporosis (10). However, which part of the proximal femur possessed the highest predictive value had been seldom discussed. Additionally, the correlation between HU values of the cortex and BMD was unclear. In addition, no study has explored the correlation between these parameters and clinical outcomes.

Thus, we attracted cortical bone thickness (CTh) and Hounsfield unit (HU) values from the cortical bone in hip computed tomography (CT) to evaluate the correlation between these parameters and BMD. This study aimed to (1) investigate the correlation between the anatomical parameters of cortical bone and BMD assessed with DXA scans; (2) calculate the sensitivity, specificity, and area under the curve (AUC) of the parameters with higher correlation to predict osteoporosis; and (3) evaluate the clinical differences between osteoporotic and nonosteoporotic patients with intertrochanteric fractures divided by the cut-off values with optimal diagnostic efficiency.

## Materials and methods

### Patient selection

**Figure 1:**
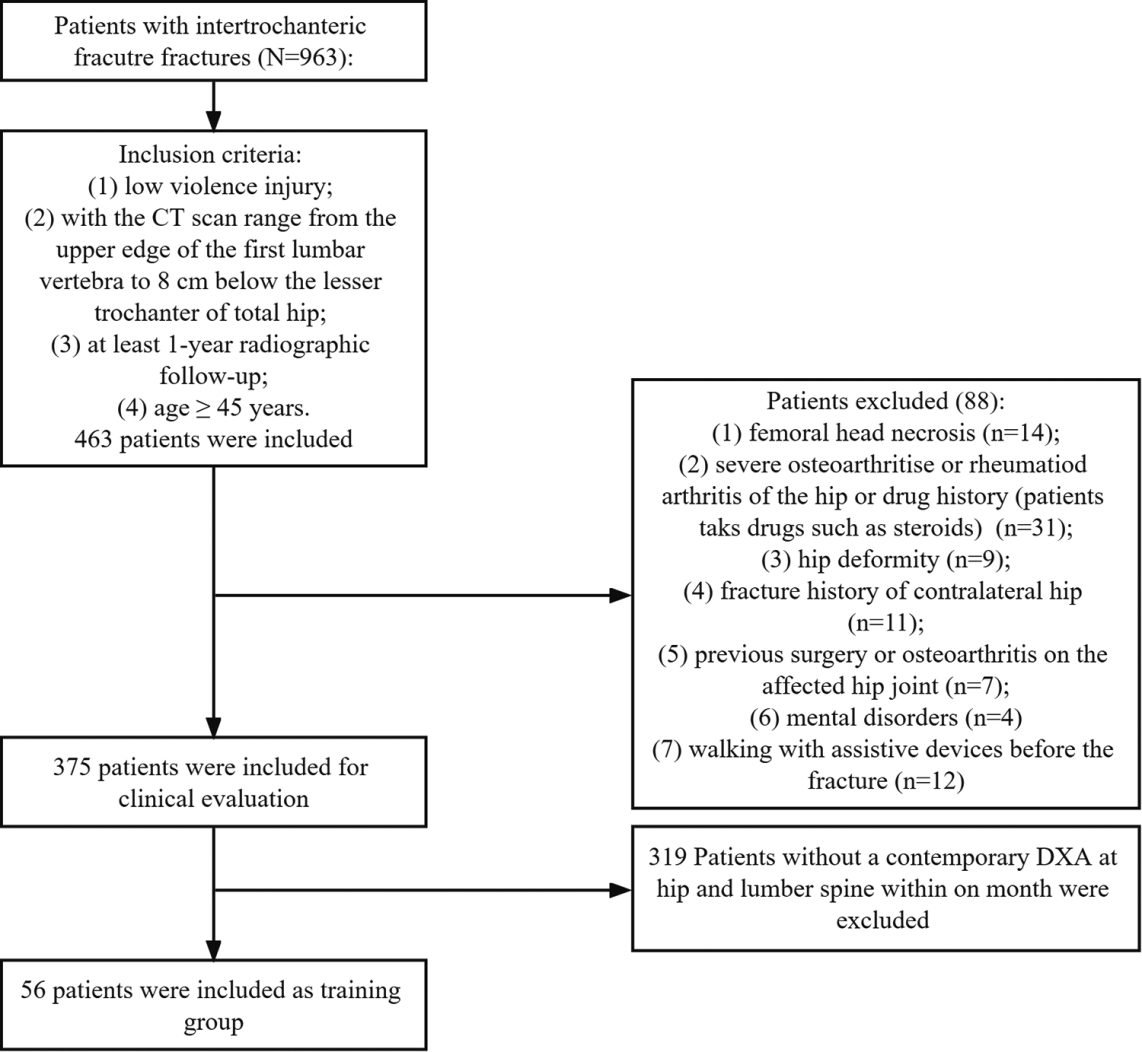
Flow chart of the inclusion and exclusion process.

Nine hundred and sixty-three patients with intertrochanteric fractures were admitted to our hospital from September 2009 to March 2017. According to the inclusion and exclusion process shown in Figure 1, 375 patients were included for clinical evaluation. Among them, 56 patients that had received CT scans from a CT machine (Siemens AG, Erlangen, Germany) and a contemporary DXA (with a Hologic Discovery system) at the unaffected hip and lumber spine at our institution within 1 month were identified for the calculation of correlation between BMD and anatomical parameters and the diagnosis efficiency of osteoporosis. This study was approved by the institutional review board (S2020-114-01).

### Region of interest for cortical bone

Patient hip CT data in the Digital Imaging and Communications in Medicine (DICOM) format were imported in Mimics 20.0 (Materialise, Belgium), and bone tissue was separated by the threshold value of 0–350 HU displaying the outline of bone clearly. Then, according to our previous study (11), 31 regions of interest (ROIs) that best reflect the stress distribution pattern in the proximal femur were defined to abstract the CTh and HU values of cortical bone.

### Femoral neck

**Figure 2:**
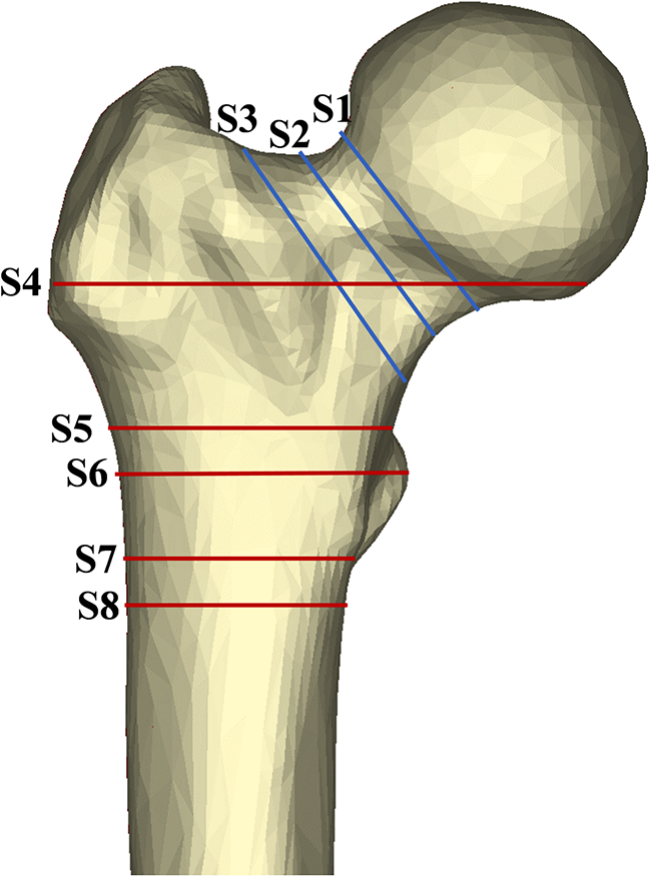
Area of exploration in proximal femoral region. The femoral neck was divided by axial S1–3 (sections at the subcephalic, middle, and bottom sites of the femoral neck, respectively). Similarly, the shaft region was divided by S4–8 (sections at 20 mm above the upper edge of the femoral lesser trochanter, the upper edge of the femoral lesser trochanter, the vertex of the femoral lesser trochanter, the lower edge of the femoral lesser trochanter, and the 20 mm below the vertex of the femoral lesser trochanter, respectively).

**Figure 3:**
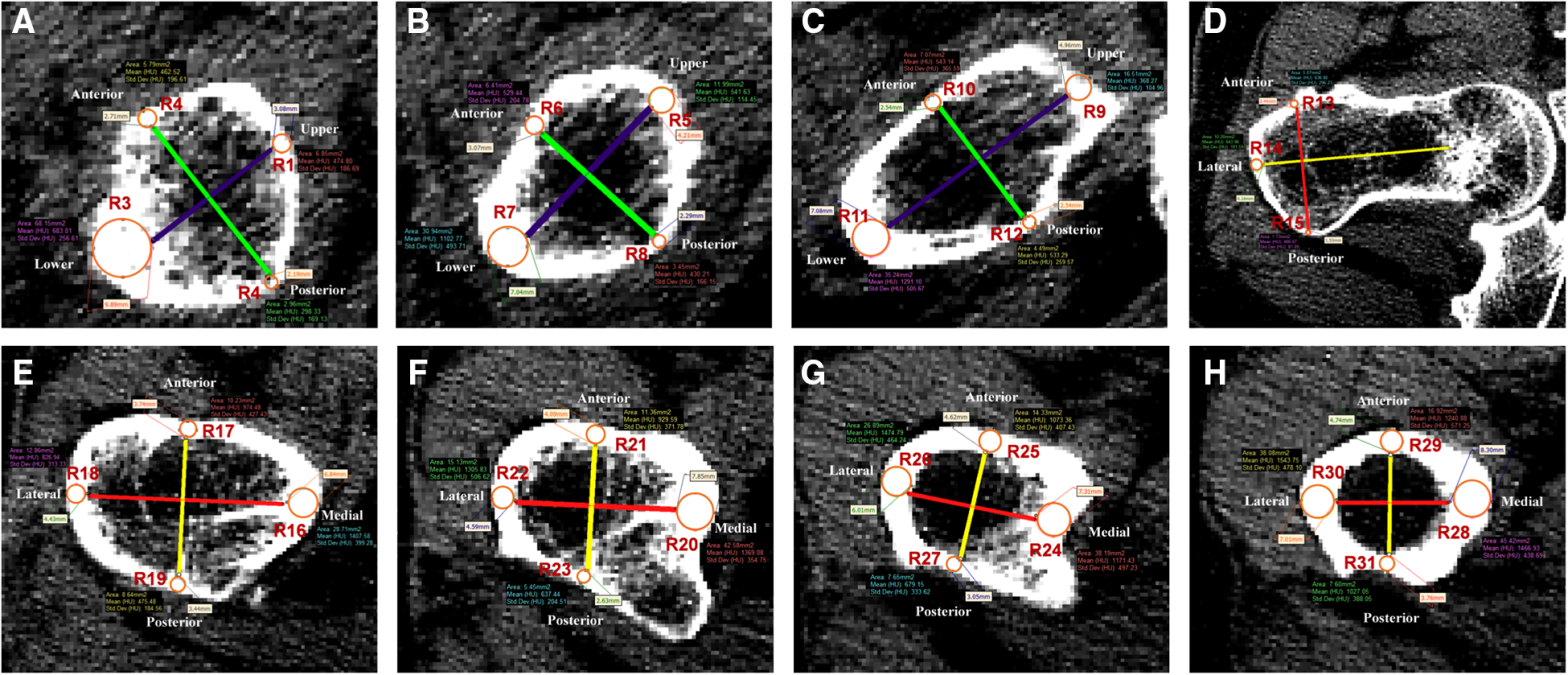
Thirty-one regions of interest were defined in the femoral neck and shaft regions. (**A–C**) Femoral neck region. Purple line was the longest diameter between the upper and lower walls of femoral neck. Green line was the longest diameter between the anterior and posterior walls of femoral neck. R1–12 were defined as the measurement points of S1–3. (**D–H**) Femoral shaft region. Red line was the longest diameter between the medial and lateral walls of the femoral shaft. Yellow line was the longest diameter between the anterior and posterior walls of the femoral shaft. R13–31 were defined as the measurement points of S4–8.

The bottom and subcephalic sites of the femoral neck were determined using the method described by Sparks et al.(12) and Zhang et al. (13). Furthermore, the middle site of the femoral neck was determined. Then, Sections 1–3 at the above sites perpendicular to the femoral neck axis were established (Figure 2). According to the method of our previous study (11), the ROIs of upper, lower, anterior, and posterior cortical bone in each section were determined as follows: the longest line between the upper and lower walls (purple line) and the longest line between the anterior and posterior walls (green line) were drawn and intersected with cortical bone, resulting in the intersections regarded as the ROIs 1–12 of cortical bone in each section of the femoral neck to extract CTh and HU values (Figures 3A–C). Then, the relevance between BMD and these parameters was analyzed.

### Femoral shaft

By the method described by Zhang et al. (14), five sections perpendicular to the femoral shaft axis were determined: S4 at 20 mm above the upper edge of the femoral lesser trochanter, S5 at the upper edge of the femoral lesser trochanter, S6 at the vertex of the femoral lesser trochanter, S7 at the lower edge of the femoral lesser trochanter, and S8 at the 20 mm below the vertex of the femoral lesser trochanter (Figure 2). According to the method of our previous study (11), in each section, the longest line (red line) between the medial and lateral walls and the longest line (yellow line) between the anterior and posterior walls were drawn to determine the ROIs 13–31 in each section of the femoral shaft to extract CTh and HU values (Figures 3D–H). Since it intersected with the femoral head, the medial wall of S4 was excluded.

### Subgroup analysis

BMD values of the lumbar spine (L1–4) and healthy hip sites were measured by a DXA scanner. According to the World Health Organization criteria: a patient with T-value at any region less than −2.5 was diagnosed with osteoporosis. Then, the sensitivity, specificity, positive predictive value, and negative predictive value of CTh and HU values in different areas for screening osteoporosis were assessed.

### Clinical evaluation

After the cut-off values with optimal diagnostic efficiency of osteoporosis were determined, 375 patients with intertrochanteric hip fracture were divided into osteoporosis and nonosteoporosis groups. The demographic information, functional parameters, and complications of different groups were collected for clinical evaluation.

### Functional parameters

Timed Up and Go (TUG) test (15), the Functional Independence Measure (FIM) (16), 2-min walk test (2MWT) (17), and Parker–Palmer scores (18) were obtained in medical records and used to assess the physical function at the final follow-up.

### Complications

Implant breakage, reduction loss, nonunion, excessive sliding, cut-out, periprosthetic fracture, infection, and loss of mobility were recorded as complications. Reduction loss was defined as a change of femoral neck-shaft angle >10°, and excessive sliding was defined as a sliding distance ≥10 mm in postoperative radiography follow-up (19).

### Reliability analysis

The intraclass correlation coefficient (ICC) was used to assess the reliability of the measurement methods established in this study. The CTh and HU values were measured twice independently at 2-week intervals by an orthopedic surgeon blinded to the result of the DXA scan. Independent measurements were performed by other two orthopedic colleagues within a 2-week period. The interobserver and intraobserver correlation coefficients were then calculated.

### Statistical analysis

Statistical analysis was conducted using the SPSS software (version 26.0, IBM, Armonk, NY, United States) and R package (3.6.3 version, statistical analysis and visualization). Student’s *t*-test was utilized to analyze the continuous data conforming to nonosteoporosis distribution, or a nonparametric rank sum test was used. Chi-square analysis or Fisher's exact test was performed for categorical variables. Pearson's correlation coefficient (*r*) was utilized to test the association between CTh value, HU value, and BMD. The receiver operating characteristic (ROC) curve was used to determine the diagnostic efficiency of the parameters of ROIs for osteoporosis. The cut-off values of variables were determined by calculating AUC. Then, sensitivity, specificity, positive predictive value, and negative predictive value were calculated. The level of statistical significance was defined as *p* < 0.05.

## Results

### Correlation between hip BMD, CTh, and HU values

Fifty-six patients (40 female and 16 male) with a mean age of 82 ± 9 years were enrolled for the calculation of Pearson's correlation coefficient and the diagnostic efficiency of osteoporosis in our study. The mean BMD values of the hip and lumbar spine were 0.57 ± 0.19 and 0.82 ± 0.16 g/cm^2^, respectively. Other characteristics are shown in Table 1.

**Table 1:** The characteristics of the patients with a DXA at the hip and lumber spine.

Characteristic
General information	Mean age, years	81.91 ± 9.05
	Gender (female/male), *n* (%)	40 (71.4)/16 (28.6)
	Femur side (right/left), *n* (%)	27 (48.2)/29 (51.8)
	Mean BMI, kg/m^2^	23 ± 3.9
Mean BMD, g/cm^2^	Hip	0.57 ± 0.19
	Lumbar spine (L1–L4)	0.82 ± 0.16
Mean T-score, g/cm^2^	Hip	−2.70 ± 1.37
	Lumbar spine (L1–L4)	−2.18 ± 1.37

DXA, dual-energy x-ray absorptiometry; BMI, body mass index; BMD, bone mineral density.

### Correlation between hip BMD and CTh values

There was a moderate correlation between hip BMD and the CTh value of ROI 16 (*r* = 0.523, *p* < 0.001), ROI 21 (*r* = 0.475, *p* < 0.001), ROI 23 (*r* = 0.501, *p* < 0.001), ROI 25 (*r* = 0.480, *p* < 0.001), and ROI 28 (*r* = 0.457, *p* = 0.001) (Table 2). The mean CTh value and the correlation between other CTh values and BMD are summarized in Supplementary Tables S1, 2.

**Table 2:** Correlation between CTh and HU values of the proximal femur and hip BMD.

Variables	Regions	Measurement	Correlation	
			*r*	*p*
CTh (mm)	ROI 16	6.01 ± 1.39	0.523	<0.001
	ROI 21	3.37 ± 0.84	0.475	<0.001
	ROI 23	2.67 ± 0.81	0.501	<0.001
	ROI 25	3.91 ± 1.03	0.480	<0.001
	ROI 28	6.39 ± 1.39	0.457	0.001
HU values (HU)	ROI 14	419.48 ± 135.38	0.445	0.001
	ROI 23	474.74 ± 122.65	0.449	0.001
	ROI 27	570.09 ± 139.75	0.481	<0.001
	ROI 29	919.90 ± 170.33	0.446	0.001

CTh, cortical bone thickness; HU, Hounsfield unit; BMD, bone mineral density; ROI, region of interest.

### Correlation between hip BMD and HU values

The moderate correlation was found between hip BMD and the HU values of ROI 14 (*r* = 0.445, *p* = 0.001), ROI 23 (*r* = 0.449, *p* = 0.001), ROI 27 (*r* = 0.481, *p* < 0.001), and ROI 29 (*r* = 0.446, *p* = 0.001) (Table 2). The mean HU value and the correlation between other HU values and BMD were summarized in Supplementary Tables S1, 2.

### Correlation between lumber BMD, CTh, and HU values

The correlations between lumber BMD, CTh, and HU values at 31 ROIs were weak, so we summarized the correlation between these parameters in 31 ROIs and lumber BMD in Supplementary Tables S1, 2.

### ROC and AUC

**Figure 4:**
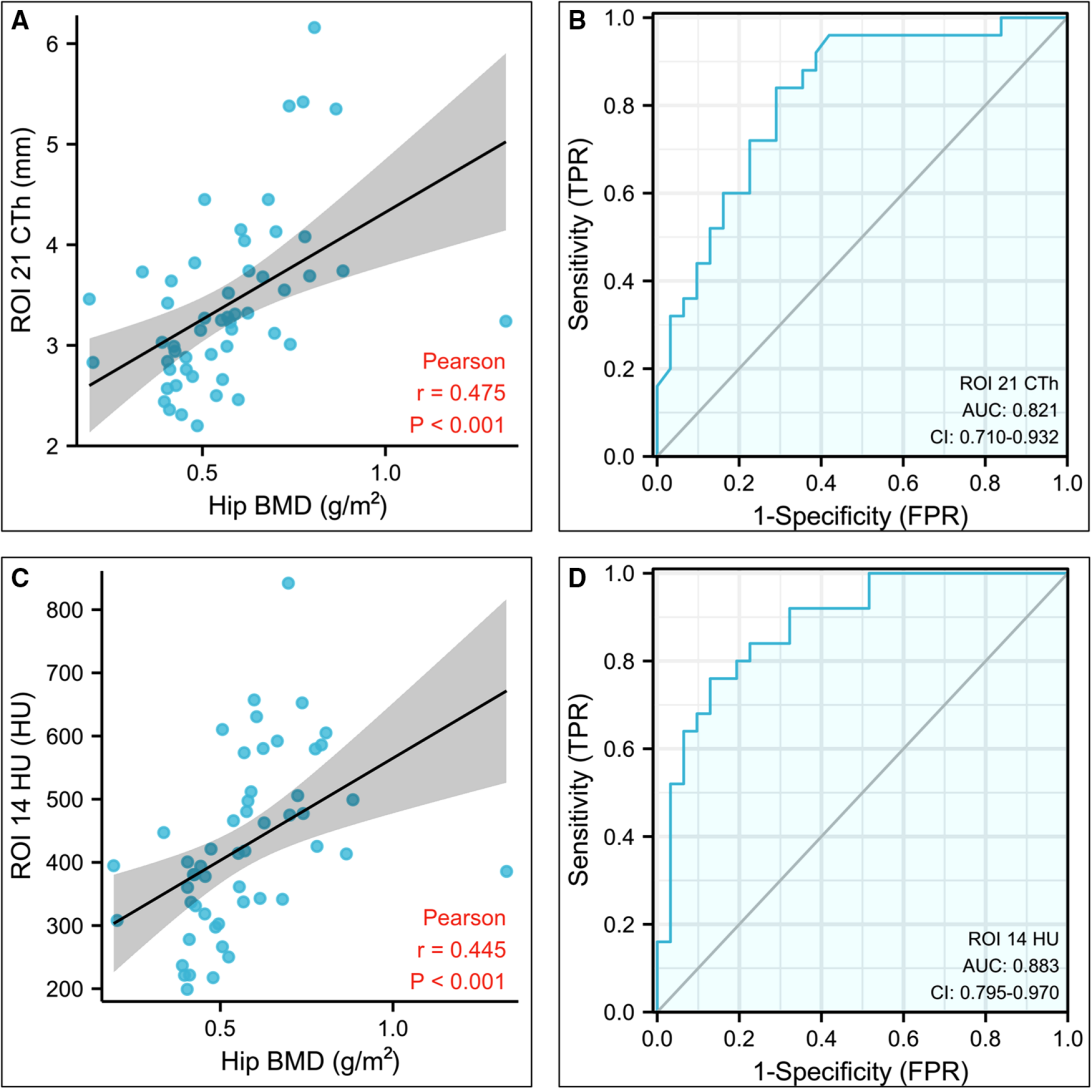
Statistical analysis results of the CTh value of the region of interest (ROI) 21 and HU value of the ROI 14. (**A**) Plot illustrating the correlation between the CTh value of ROI 21 and hip bone mineral density. (**B**) Diagnostic efficiency of the CTh value of ROI 21 for osteoporosis. (**C**) Plot illustrating the correlation between the HU value of ROI 14 and hip bone mineral density. (**D**) Diagnostic efficiency of the HU value of ROI 14 for osteoporosis. CTh, cortical bone thickness; HU, Hounsfield unit; ROI, region of interest.

The CTh and HU values with greater *r* values were used to evaluate ROC and AUC. Among them, the CTh of ROI 21 and HU of ROI 14 showed superior diagnostic accuracy for screening osteoporosis, as AUC were 0.82 (0.71– 0.93) and 0.88 (0.80– 0.97), respectively (Figure 4). The cut-off value for the CTh value of ROI 21 was 3.19 mm, with a sensitivity of 84% and a specificity of 71%. The cut-off value for the HU value of ROI 14 was 424.97 HU, with a sensitivity of 76% and a specificity of 87% (Table 3). Other CTh values of 18 ROIs and HU values of 10 ROIs were confirmed to be weaker diagnostic efficiency (displayed in Supplementary Table S3, 4).

**Table 3:** Diagnostic efficiency of ROI 21 CTh and ROI 14 HU value for osteoporosis.

Parameters	Diagnosis for nonosteoporosis						
	AUC	95% CI	Cut-off	Se	Sp	PV+	PV−
ROI 21 CTh	0.821	0.710–0.932	3.185	0.84	0.71	0.7	0.846
ROI 14 HU	0.883	0.795–0.970	424.97	0.76	0.871	0.826	0.818

ROI, region of interest; CTh, cortical bone thickness; HU, Hounsfield unit; AUC, area under the curve; Se, sensitivity; Sp, specificity; PV+, positive predictive value; PV−, negative predictive value.

### Clinical evaluation

Three hundred and seventy-five patients (240 female and 135 male) with a mean age of 77.81 ± 9.44 years were included for clinical evaluation. Their demographic information is shown in Table 5. Based on the cut-off value of CTh value at ROI 21, 375 patients were divided into the osteoporosis group (*n* = 180) and the nonosteoporosis group (*n* = 195). Based on the cut-off value of 424.97 HU at ROI 14, 375 patients were divided into the osteoporosis group (*n* = 183) and the nonosteoporosis group (*n* = 192). There was a correlation between the two diagnostic methods of osteoporosis, but there was no difference between them (Table 4). The incidence of postoperative complications in the osteoporosis group was higher than that in the nonosteoporosis group divided by the CTh value at ROI 21 (*p* < 0.05) (Table 5). The number of patients who got FIM parameters and Parker–Palmer scores is shown in Table 6. The proportion of patients completing the TUG and 2MWT is shown in Table 7. The FIM parameters and Parker–Palmer scores of the nonosteoporosis group were higher than those of the osteoporosis group divided by the HU value of ROI 14 (*p* < 0.05) (Table 6).

**Table 4:** Correlation and difference analysis of two diagnostic methods based on the cut-off values of ROI 21 CTh and ROI 14 HU value for osteoporosis.

Method	Statistics	*p* value
Fisher's test	23.198	0.000
McNemar's test	0.858	0.4

ROI, region of interest; CTh, cortical bone thickness; HU, Hounsfield unit.

**Table 5:** Patient demographics and complications by the group.

Parameters		ROI 21 CTh (mm)		P1 value	ROI 14 HU value (HU)		P2 value
		≥3.19 (*n* = 180)	<3.19 (*n* = 195)		≥424.97 (*n* = 192)	<424.97 (*n* = 183)	
Sex	Female	95	145	<0.001	106	134	<0.001
	Male	85	50		86	49	
Side	Left	88	103	0.447	97	94	0.870
	Right	92	92		95	89	
Age (year)		76.48 ± 10.22	79.04 ± 8.50	0.009	76.26 ± 10.43	79.45 ± 7.98	0.001
Height (cm)		164.71 ± 8.68	160.71 ± 7.40	<0.001	164.29 ± 8.32	160.89 ± 7.87	<0.001
Weight (kg)		64.24 ± 12.27	57.85 ± 11.27	<0.001	63.70 ± 12.42	58.00 ± 11.23	<0.001
BMI (kg/m^2^)		23.60 ± 3.77	22.35 ± 3.84	0.002	23.52 ± 3.86	22.35 ± 3.76	0.003
AO classification, *n*	A1	60	56	0.568	59	57	0.992
	A2	100	119		113	106	
	A3	20	21		22	21	
Fixation type	Gamma3	6	11	0.562	11	6	0.088
	Intertan	17	18		23	12	
	PFNA	157	166		158	165	
Complications, *n*		12	24	0.037	18	18	0.880
	Loss of reduction	3	0		2	1	
	Excessive sliding of the cephalic nail	1	4		3	2	
	Cut-out	0	3		1	2	
	Implant breakage	3	1		3	1	
	Nonunion	0	1		1	0	
	Infection	1	2		3	0	
	Periprosthetic fracture	0	4		2	2	
	Loss of mobility	2	5		1	6	
	Contralateral hip fracture	2	4		2	4	

ROI, region of interest; CTh, cortical bone thickness; HU, Hounsfield unit; BMI, body mass index; AO, arbeitsgemeinschaftfür osteosynthesefragen; PFNA, proximal femoral nail antirotation.

**Table 6:** Functional ability by the group.

		Functional independence measure	Parker–Palmer score
Group	Numbers that got the parameters, *n* (%)	Mean ± SD	Mean ± SD
ROI 21 CTh (mm)			
≥3.19 (*n* = 180)	148 (82.2%)	97.57 ± 20.00	6.76 ± 2.13
<3.19 (*n* = 195)	164 (84.1%)	96.07 ± 17.32	6.84 ± 1.88
P1 value	0.627	0.483	0.731
ROI 14 HU value			
≥ 424.97 (*n* = 192)	159 (82.8%)	99.23 ± 16.79	7.02 ± 1.87
<424.97 (*n* = 183)	153 (83.6%)	94.24 ± 20.09	6.57 ± 2.11
P2 value	0.837	0.018	0.047

ROI, region of interest; CTh, cortical bone thickness; HU, Hounsfield unit.

**Table 7:** Performance-based functional ability by the group measured using the 2-min walk test and Timed Up and Go test.

	2-min Walk Test (m)		Timed Up and Go Test (s)	
Group	Proportion who completed test, *n* (%)	Mean ± SD	Proportion who completed test, *n* (%)	Mean ± SD
ROI 21 CTh (mm)				
≥3.19 (*n* = 180)	136 (75.6%)	67.03 ± 24.08	139 (77.2%)	18.70 ± 10.60
<3.19 (*n* = 195)	155 (79.5%)	62.15 ± 21.87	156 (80.0%)	18.47 ± 7.56
P1 value	0.362	0.071	0.512	0.834
ROI 14 HU value				
≥ 424.97 (*n* = 192)	151 (78.6%)	66.72 ± 22.59	154 (80.2%)	19.09 ± 8.85
<424.97 (*n* = 183)	140 (76.5%)	61.96 ± 23.30	141 (77.0%)	18.11 ± 9.34
P2 value	0.619	0.079	0.455	0.356

ROI, region of interest; CTh, cortical bone thickness; HU, Hounsfield unit.

### The reliability of measurement

**Figure 5:**
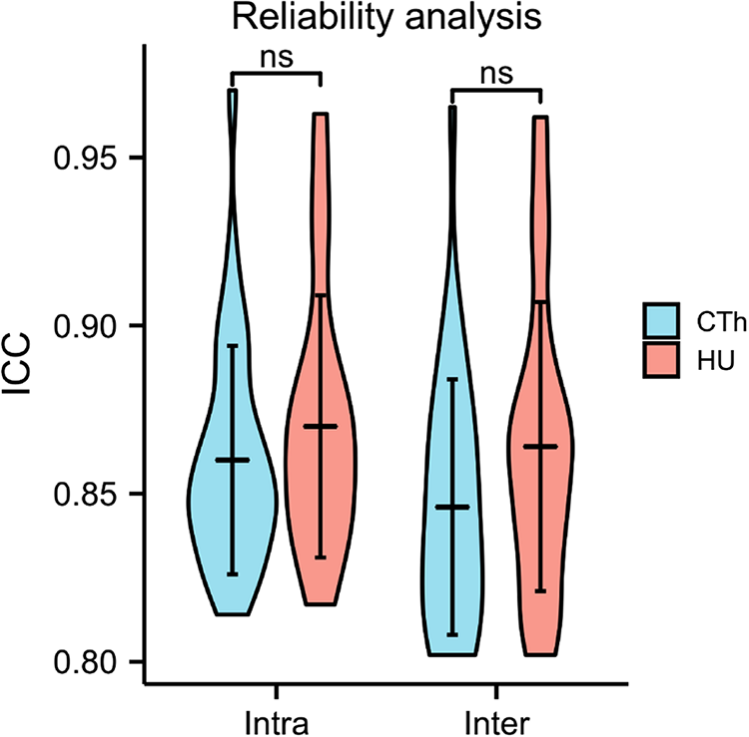
Rater reliability analysis.

The intraclass correlation coefficient (ICC) was 0.803–0.965 for interobserver reliability and 0.814–0.970 for intraobserver reliability (Figure 5; Supplementary Figures S1–4).

### Discussion

Our research reflected that CTh and HU in the exact areas of the proximal femur could be reliable indexes for screening osteoporosis, verifying that BMD could be assessed from other image data. The above two methods had the same diagnostic rate for osteoporosis and could replace each other (Table 4). And osteoporosis and nonosteoporosis groups divided by the above cut-off values displayed distinct differences in clinical outcomes. Meanwhile, the measurement methods of CTh and HU values showed excellent interrater and intrarater reliability. Therefore, our study provided clinicians with an effective and convenient method for screening osteoporosis and predicting clinical outcomes.

In this study, we found that the CTh and HU values of the cortex in the proximal femur pronouncedly corresponded with hip BMD but were weakly associated with lumbar spine BMD. This could be explained by the fact that these anatomic parameters were extracted from the hip not the lumbar spine. Furthermore, BMD of hip DXA correlated better with these parameters in the intertrochanteric region (ROIs 13–27) than those in the femoral neck (ROIs 1–12) and shaft (ROIs 28–31) regions, suggesting that the CTh and HU values of the intertrochanteric region were more suitable for predicting bone quality. We believed that this difference mainly resulted from the intertrochanteric region, as the separation zone between the cortical and cancellous bone of the proximal femur, was more sensitive to deterioration of bone quality. In addition, Tang et al. (11, 20–22) proposed the “triangular stability theory” of the proximal femur and pointed out that the proximal femur was stabilized by a structural mechanical model formed by the medial, lateral, and upper sides. The ROI 14 was located at the cortex of the vastus lateralis ridge, which was the junction between the lateral and upper sides. The ROI 21 was located at the anterior wall at the vertex of the lesser trochanter, which was the junction between the lateral and medial sides. Both regions could effectively predict BMD and mechanical stability and might prevent the occurrence of osteoporotic fractures and postoperative complications.

Clinically, a simple and reliable method for screening osteoporosis was vital for the early prevention of osteoporotic fracture and the optimal choice of surgical scheme. Therefore, the concept of “opportunistic osteoporosis screening” was proposed and popularized (23). Mather et al. (24), Patterson et al. (25), and Ye et al. (26) analyzed the correlation between BMD and the cortical bone thickness of proximal humerus, distal tibia, and distal radius on x-ray, respectively, and verified that the cortical bone thickness of different sites could be used to screen osteoporosis. Wagner et al. (27) and Schreiber et al. (23) measured the HU values of cancellous portions of the distal ulna and radius from wrist CT scans to glean additional information for predicting bone quality. Then, Ehresman et al. (28) utilized a novel MRI-based score, the Vertebral Bone Quality score, to predict osteoporosis and proposed this score to be a significant predictor of osteoporosis with an accuracy of 81%. Compared with the above previous studies, a strength of this study was that hip CT of femoral intertrochanteric fracture was a routine examination and accurately reflected a person's true anatomy. Another strength of this study was that these measurement parameters were all abstracted from the hip, which was the site with the most serious consequences of osteoporotic fractures, such as femoral neck and intertrochanteric fractures (29). To our knowledge, no study has conducted a correlation analysis between these parameters and BMD. Thus, our method would provide the clinician with a novel prospect to assess BMD.

Meanwhile, our study evaluated the clinical difference between osteoporosis and nonosteoporosis groups divided by the cut-off values. When the group was divided by the cut-off value of CTh in ROI 21, the osteoporotic group occupied a higher incidence of complications. Due to reduced BMD and microarchitectural deterioration, the mechanical strength of osteoporotic cancellous bone significantly decreased, resulting in that osteoporotic bone could not resist normal screw pullout strength and insertional torque (4, 30). When the normal load was applied to osteoporotic cancellous bone, the proximal femur would have the tendency of varus, and the stress between implant and bone tended to be concentrated, leading to a higher incidence of complications (30). Previous studies also confirmed that patients with osteoporotic fractures occupied a higher rate of complications (31). However, there was no statistical difference in the incidence of complications between different groups divided by the cut-off value of HU in ROI 14 (*p* > 0.05), which might result from the lower sensitivity of the cut-off value of HU in ROI 14 led to more misdiagnosis of actual osteoporotic patients. Thus, the cut-off value of CTh in ROI 21 was suggested as the optimal index for predicting clinical outcomes. After the osteoporotic patients were selected, the initiation of osteoporotic treatment should be considered for preventing the happen of osteoporotic fractures (32). Also, some implants with better mechanical stability, such as medial sustain nail based on femoral proximal triangular stability theory (33), should be adopted to prevent the happening of complications.

Our study had some limitations. First, the sample size was small, but this would not likely have dramatic effects on our results since the excellent measuring reliability and significant results. Second, this study only involved the patient experiencing intertrochanteric fracture, so the result of the study might not apply to the general population. Third, only Chinese was selected in our study and the result might not be generalizable to all races. Despite these limitations, this study provided a useful method for screening for osteoporosis and provided guidance for clinical treatment.

In conclusion, the CTh of ROI 21 and HU value of ROI 14 were simple and effective screening indexes to predict BMD, which could help clinicians prevent the happen of osteoporotic fractures and postoperative complications.

## Data Availability

The raw data supporting the conclusions of this article will be made available by the authors, without undue reservation.
