# Supplementary material for: Prediction of osteoporosis from proximal femoral cortical bone thickness and Hounsfield unit value with clinical significance
Source: Front Surg. 2023 Jan 6;9:1047603. doi: 10.3389/fsurg.2022.1047603 (PMC9852615; doi:10.3389/fsurg.2022.1047603)
Supplement: Supplementary file 1 [file Datasheet1.docx]

**Appendix table 1.** Correlation between CTh values and the BMD results of hip and lumbar spine measured by DXA

| ROIs | CTh  (mm, M±SD) | Correlation | | | |
| --- | --- | --- | --- | --- | --- |
|  |  | Hip BMD | | Lumbar spine BMD | |
|  |  | r | p | r | p |
| ROI 1 | 2.99 ± 0.93 | 0.376 | 0.006 | 0.122 | 0.383 |
| ROI 2 | 2.18 ± 0.56 | 0.207 | 0.142 | 0.082 | 0.557 |
| ROI 3 | 7.76 ± 2.22 | 0.362 | 0.008 | 0.347 | 0.011 |
| ROI 4 | 2.07 ± 0.50 | 0.211 | 0.133 | 0.013 | 0.927 |
| ROI 5 | 3.33 ± 0.96 | 0.188 | 0.183 | 0.05 | 0.722 |
| ROI 6 | 2.41 ± 0.68 | 0.322 | 0.02 | 0.08 | 0.567 |
| ROI 7 | 6.29 ± 1.49 | 0.33 | 0.017 | 0.153 | 0.274 |
| ROI 8 | 2.11 ± 0.56 | 0.42 | 0.002 | 0.07 | 0.619 |
| ROI 9 | 4.27 ± 1.28 | 0.176 | 0.212 | 0.199 | 0.153 |
| ROI 10 | 2.48 ± 0.64 | 0.094 | 0.508 | -0.101 | 0.471 |
| ROI 11 | 5.96 ± 1.52 | 0.43 | 0.001 | 0.169 | 0.227 |
| ROI 12 | 2.25 ± 0.53 | 0.123 | 0.383 | 0.066 | 0.64 |
| ROI 13 | 2.40 ± 0.54 | 0.175 | 0.214 | 0.211 | 0.13 |
| ROI 14 | 3.48 ± 0.99 | 0.302 | 0.03 | 0.343 | 0.012 |
| ROI 15 | 1.98 ± 0.54 | 0.2 | 0.155 | -0.016 | 0.908 |
| ROI 16 | 6.01 ± 1.39 | 0.523 | <0.001 | 0.294 | 0.033 |
| ROI 17 | 3.12 ± 0.73 | 0.291 | 0.036 | 0.187 | 0.181 |
| ROI 18 | 3.74 ± 1.57 | 0.376 | 0.006 | 0.133 | 0.341 |
| ROI 19 | 2.23 ± 0.56 | 0.392 | 0.004 | 0.111 | 0.431 |
| ROI 20 | 7.27 ± 1.87 | 0.268 | 0.055 | 0.597 | <0.001 |
| ROI 21 | 3.37 ± 0.84 | 0.475 | <0.001 | 0.29 | 0.035 |
| ROI 22 | 4.51 ± 1.33 | 0.375 | 0.006 | 0.06 | 0.669 |
| ROI 23 | 2.67 ± 0.81 | 0.501 | <0.001 | 0.13 | 0.354 |
| ROI 24 | 6.75 ± 1.54 | 0.315 | 0.023 | 0.401 | 0.003 |
| ROI 25 | 3.91 ± 1.03 | 0.48 | <0.001 | 0.247 | 0.074 |
| ROI 26 | 5.06 ± 1.06 | 0.382 | 0.005 | 0.254 | 0.066 |
| ROI 27 | 3.22 ± 0.92 | 0.439 | 0.001 | 0.249 | 0.072 |
| ROI 28 | 6.39 ± 1.39 | 0.457 | 0.001 | 0.299 | 0.029 |
| ROI 29 | 4.43 ± 1.00 | 0.404 | 0.003 | 0.151 | 0.28 |
| ROI 30 | 5.81 ± 1.07 | 0.321 | 0.02 | 0.183 | 0.19 |
| ROI 31 | 3.67 ± 0.92 | 0.409 | 0.003 | 0.253 | 0.068 |

BMD, bone mineral density; DXA, dual energy X-ray absorptiometry; CTh, cortical bone thickness; ROI, region of interest;

**Appendix table 2.** Correlation between cortical bone HU values and the BMD results of hip and lumbar spine measured by DXA

| ROIs | Hounsfield unit (HU, M±SD) | Correlation | | | |
| --- | --- | --- | --- | --- | --- |
|  |  | Hip BMD | | Lumbar spine BMD | |
|  |  | r | p | r | p |
| ROI 1 | 403.15 ± 81.46 | 0.363 | 0.008 | 0.245 | 0.077 |
| ROI 2 | 321.87 ± 92.01 | 0.249 | 0.075 | 0.253 | 0.067 |
| ROI 3 | 662.39 ± 128.09 | 0.283 | 0.042 | -0.039 | 0.783 |
| ROI 4 | 305.75 ± 85.16 | 0.228 | 0.105 | 0.099 | 0.481 |
| ROI 5 | 423.72 ± 88.52 | 0.22 | 0.117 | 0.038 | 0.785 |
| ROI 6 | 402.31 ± 102.79 | 0.397 | 0.004 | 0.224 | 0.107 |
| ROI 7 | 955.50 ± 154.40 | 0.258 | 0.064 | -0.17 | 0.223 |
| ROI 8 | 361.63 ± 89.93 | 0.131 | 0.355 | 0.1 | 0.476 |
| ROI 9 | 411.07 ± 149.02 | 0.071 | 0.617 | 0.071 | 0.612 |
| ROI 10 | 464.01 ± 105.87 | 0.352 | 0.01 | 0.119 | 0.394 |
| ROI 11 | 1045.70 ± 165.07 | 0.173 | 0.219 | 0.157 | 0.262 |
| ROI 12 | 422.49 ± 103.38 | 0.076 | 0.593 | 0.111 | 0.428 |
| ROI 13 | 421.18 ± 114.88 | 0.276 | 0.048 | 0.184 | 0.186 |
| ROI 14 | 419.48 ± 135.38 | 0.445 | 0.001 | 0.457 | 0.001 |
| ROI 15 | 267.47 ± 90.86 | 0.274 | 0.05 | 0.322 | 0.019 |
| ROI 16 | 1064.24 ± 135.58 | 0.112 | 0.43 | 0.1 | 0.476 |
| ROI 17 | 650.83 ± 131.78 | 0.268 | 0.054 | 0.31 | 0.024 |
| ROI 18 | 665.88 ± 133.90 | 0.187 | 0.184 | 0.1 | 0.487 |
| ROI 19 | 371.39 ± 116.27 | 0.326 | 0.018 | 0.18 | 0.196 |
| ROI 20 | 1034.49 ± 169.59 | 0.251 | 0.073 | 0.213 | 0.125 |
| ROI 21 | 721.13 ± 152.09 | 0.399 | 0.003 | 0.232 | 0.094 |
| ROI 22 | 915.26 ± 169.44 | 0.334 | 0.016 | 0.147 | 0.295 |
| ROI 23 | 474.74 ± 122.65 | 0.449 | 0.001 | 0.382 | 0.005 |
| ROI 24 | 1096.86 ± 159.67 | 0.276 | 0.048 | 0.178 | 0.203 |
| ROI 25 | 837.86 ± 157.11 | 0.402 | 0.003 | 0.206 | 0.139 |
| ROI 26 | 1100.27 ± 150.03 | 0.221 | 0.116 | 0.179 | 0.2 |
| ROI 27 | 570.09 ± 139.75 | 0.481 | <0.001 | 0.379 | 0.005 |
| ROI 28 | 1152.76 ± 164.05 | 0.313 | 0.024 | 0.223 | 0.108 |
| ROI 29 | 919.90 ± 170.33 | 0.446 | 0.001 | 0.188 | 0.178 |
| ROI 30 | 1184.17 ± 147.49 | 0.243 | 0.082 | 0.246 | 0.076 |
| ROI 31 | 702.75 ± 172.12 | 0.382 | 0.005 | 0.266 | 0.055 |

BMD, bone mineral density; DXA, dual energy X-ray absorptiometry; HU, Hounsfield unit; ROI, region of interest

**Appendix table 3.** Diagnostic efficiency of CTh values of the proximal femur for osteoporosis (AUC>0.70).

| ROIs | Diagnostic ability for osteoporosis | | | | | | |
| --- | --- | --- | --- | --- | --- | --- | --- |
|  | AUC | 95% CI | Cut-off | Se | Sp | PV+ | PV- |
| ROI 3 | 0.725 | 0.578-0.871 | 8.63 | 0.52 | 0.968 | 0.929 | 0.714 |
| ROI 11 | 0.746 | 0.611-0.882 | 6.445 | 0.6 | 0.903 | 0.833 | 0.737 |
| ROI 14 | 0.739 | 0.603-0.874 | 3.115 | 0.88 | 0.613 | 0.647 | 0.864 |
| ROI 16 | 0.735 | 0.602-0.868 | 5.915 | 0.76 | 0.645 | 0.633 | 0.769 |
| ROI 18 | 0.715 | 0.580-0.851 | 3.225 | 0.84 | 0.581 | 0.618 | 0.818 |
| ROI 19 | 0.708 | 0.570-0.846 | 2.505 | 0.44 | 0.903 | 0.786 | 0.667 |
| ROI 20 | 0.763 | 0.634-0.891 | 7.425 | 0.68 | 0.774 | 0.708 | 0.75 |
| ROI 21 | 0.821 | 0.710-0.932 | 3.185 | 0.84 | 0.71 | 0.7 | 0.846 |
| ROI 22 | 0.712 | 0.570-0.853 | 4.295 | 0.8 | 0.677 | 0.667 | 0.808 |
| ROI 23 | 0.766 | 0.638-0.894 | 2.31 | 0.88 | 0.548 | 0.611 | 0.85 |
| ROI 24 | 0.726 | 0.593-0.860 | 6.55 | 0.76 | 0.645 | 0.633 | 0.769 |
| ROI 25 | 0.788 | 0.662-0.914 | 4.485 | 0.52 | 1 | 1 | 0.721 |
| ROI 26 | 0.709 | 0.569-0.849 | 4.9 | 0.84 | 0.645 | 0.656 | 0.833 |
| ROI 27 | 0.746 | 0.616-0.877 | 3.735 | 0.44 | 0.935 | 0.846 | 0.674 |
| ROI 28 | 0.773 | 0.651-0.894 | 6.205 | 0.8 | 0.677 | 0.667 | 0.808 |
| ROI 29 | 0.716 | 0.58-0.853 | 2.055 | 0.68 | 0.677 | 0.63 | 0.724 |
| ROI 30 | 0.707 | 0.566-0.848 | 4.845 | 0.52 | 0.871 | 0.765 | 0.692 |
| ROI 31 | 0.709 | 0.557-0.861 | 4.205 | 0.56 | 0.903 | 0.824 | 0.718 |

CTh, cortical bone thickness; ROI, region of interest

**Appendix table 4.** Diagnostic efficiency of cortical bone Hounsfield unit (HU) values of the proximal femur for osteoporosis (AUC>0.70).

| ROIs | Diagnostic ability for osteoporosis | | | | | | |
| --- | --- | --- | --- | --- | --- | --- | --- |
|  | AUC | 95% CI | Cut-off | Se | Sp | PV+ | PV- |
| ROI 14 | 0.883 | 0.795-0.970 | 424.965 | 0.76 | 0.871 | 0.826 | 0.818 |
| ROI 17 | 0.712 | 0.577-0.848 | 606.99 | 0.84 | 0.581 | 0.618 | 0.818 |
| ROI 19 | 0.732 | 0.599-0.864 | 376.32 | 0.68 | 0.742 | 0.68 | 0.742 |
| ROI 21 | 0.728 | 0.593-0.863 | 802.34 | 0.6 | 0.839 | 0.75 | 0.722 |
| ROI 23 | 0.754 | 0.623-0.884 | 474.09 | 0.8 | 0.645 | 0.645 | 0.8 |
| ROI 24 | 0.706 | 0.564-0.848 | 1049.56 | 0.88 | 0.548 | 0.611 | 0.85 |
| ROI 25 | 0.763 | 0.632-0.893 | 873.38 | 0.72 | 0.839 | 0.783 | 0.788 |
| ROI 27 | 0.779 | 0.657-0.902 | 634.35 | 0.6 | 0.871 | 0.789 | 0.73 |
| ROI 29 | 0.759 | 0.629-0.888 | 862.295 | 0.88 | 0.581 | 0.629 | 0.857 |
| ROI 31 | 0.761 | 0.635-0.887 | 624.63 | 0.88 | 0.613 | 0.647 | 0.864 |

HU, Hounsfield unit; ROI, region of interest


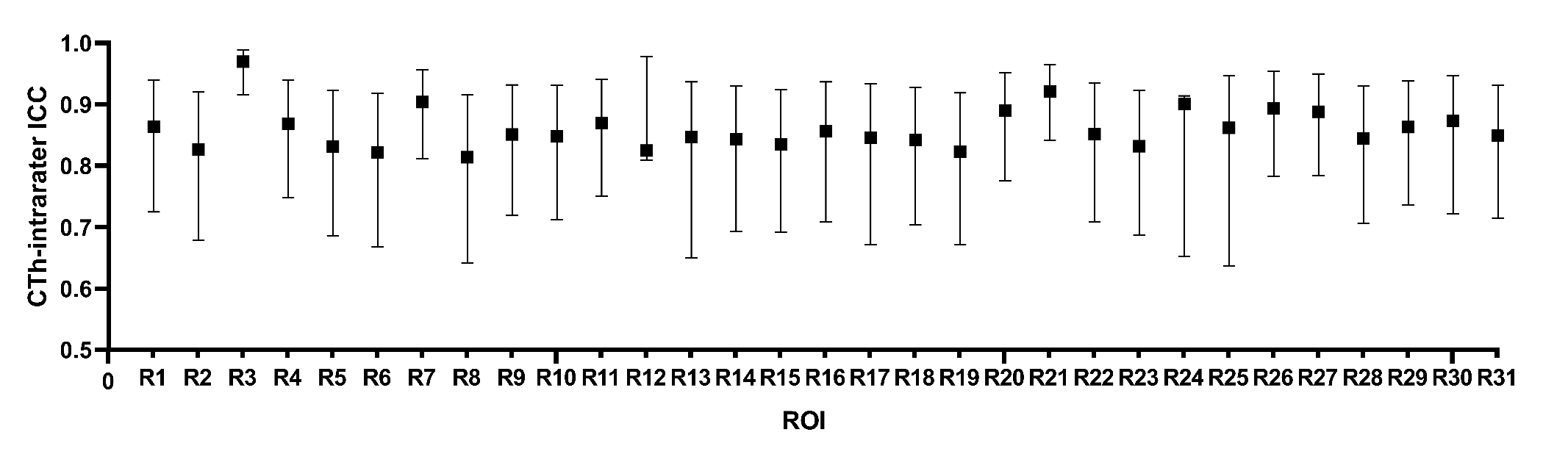


**Appendix** **Fig.1** Intrarater Reliability for cortical bone thickness (CTh) value in 31 regions of interest (ROI).


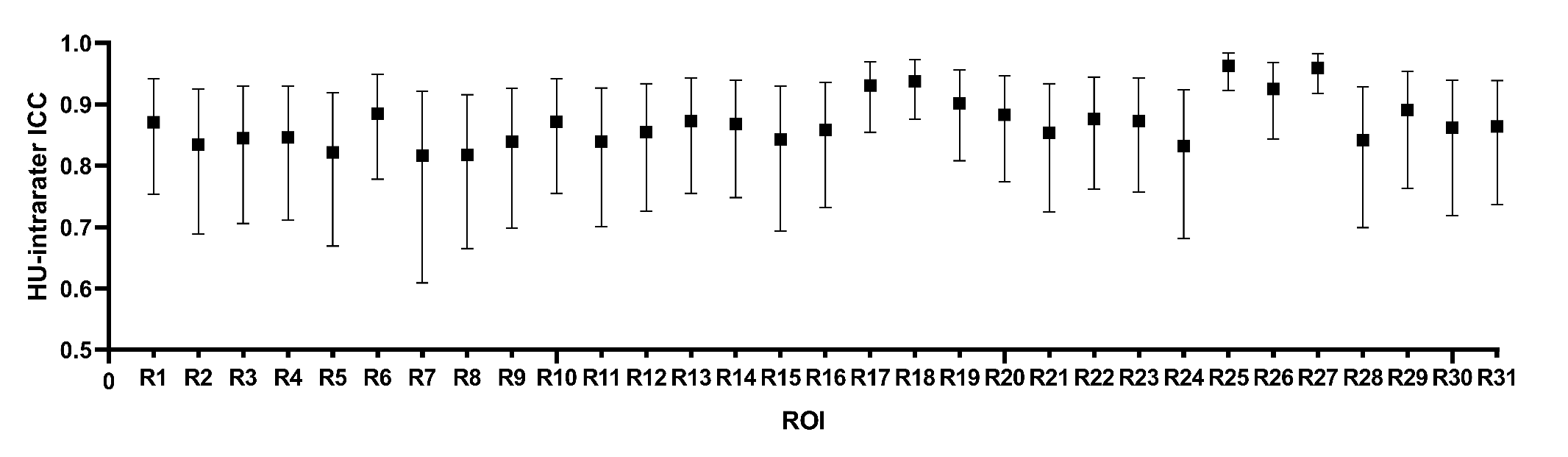


**Appendix** **Fig.2** Intrarater Reliability for Hounsfield unit (HU) value in 31 regions of interest (ROI).


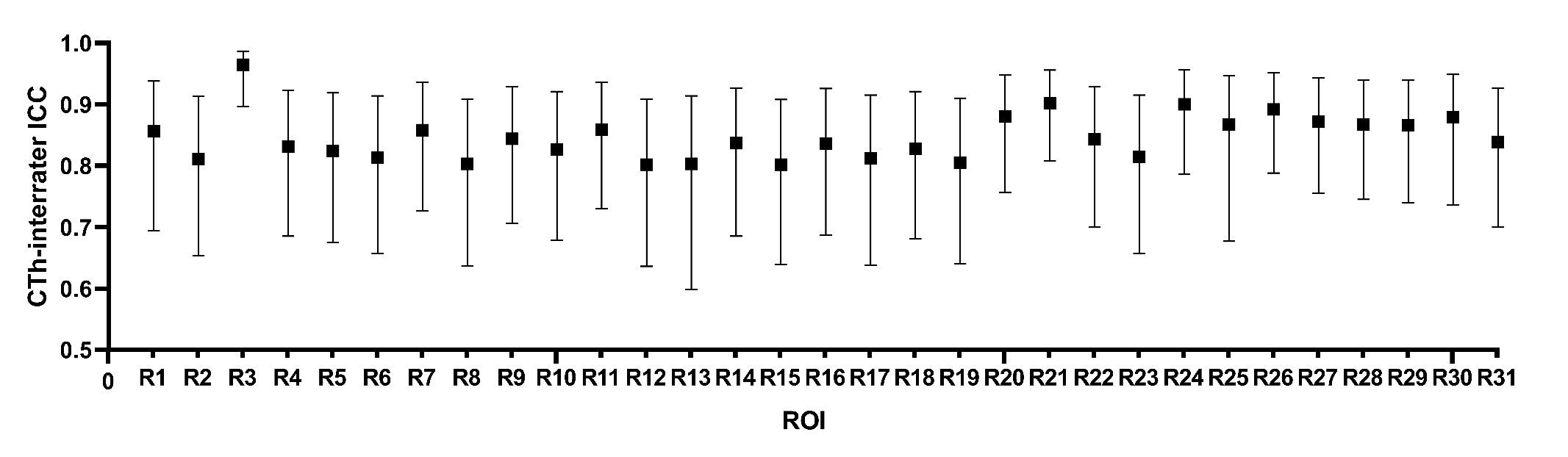


**Appendix** **Fig.3** Interrater Reliability for cortical bone thickness (CTh) value in 31 regions of interest (ROI).


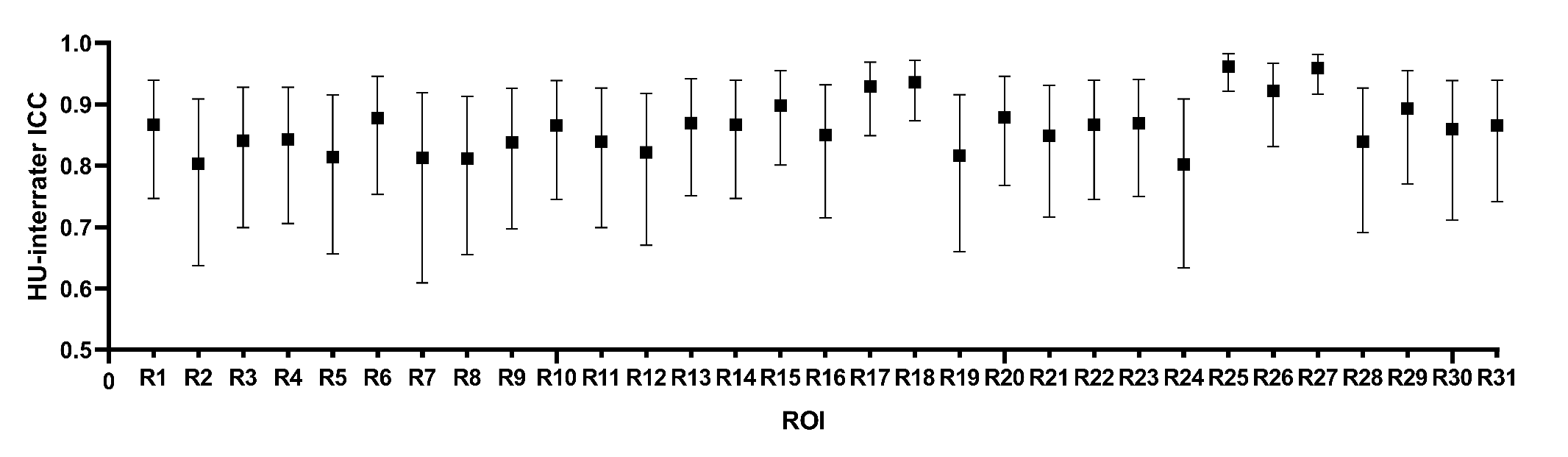


**Appendix** **Fig.4** Interrater Reliability for Hounsfield unit (HU) value in 31 regions of interest (ROI).
